# Supplementary figures and images for: Real-time estimation of disease activity in emerging outbreaks using internet search information
Source: PLoS Comput Biol. 2020 Aug 17;16(8):e1008117. doi: 10.1371/journal.pcbi.1008117 (PMC7451983; doi:10.1371/journal.pcbi.1008117)

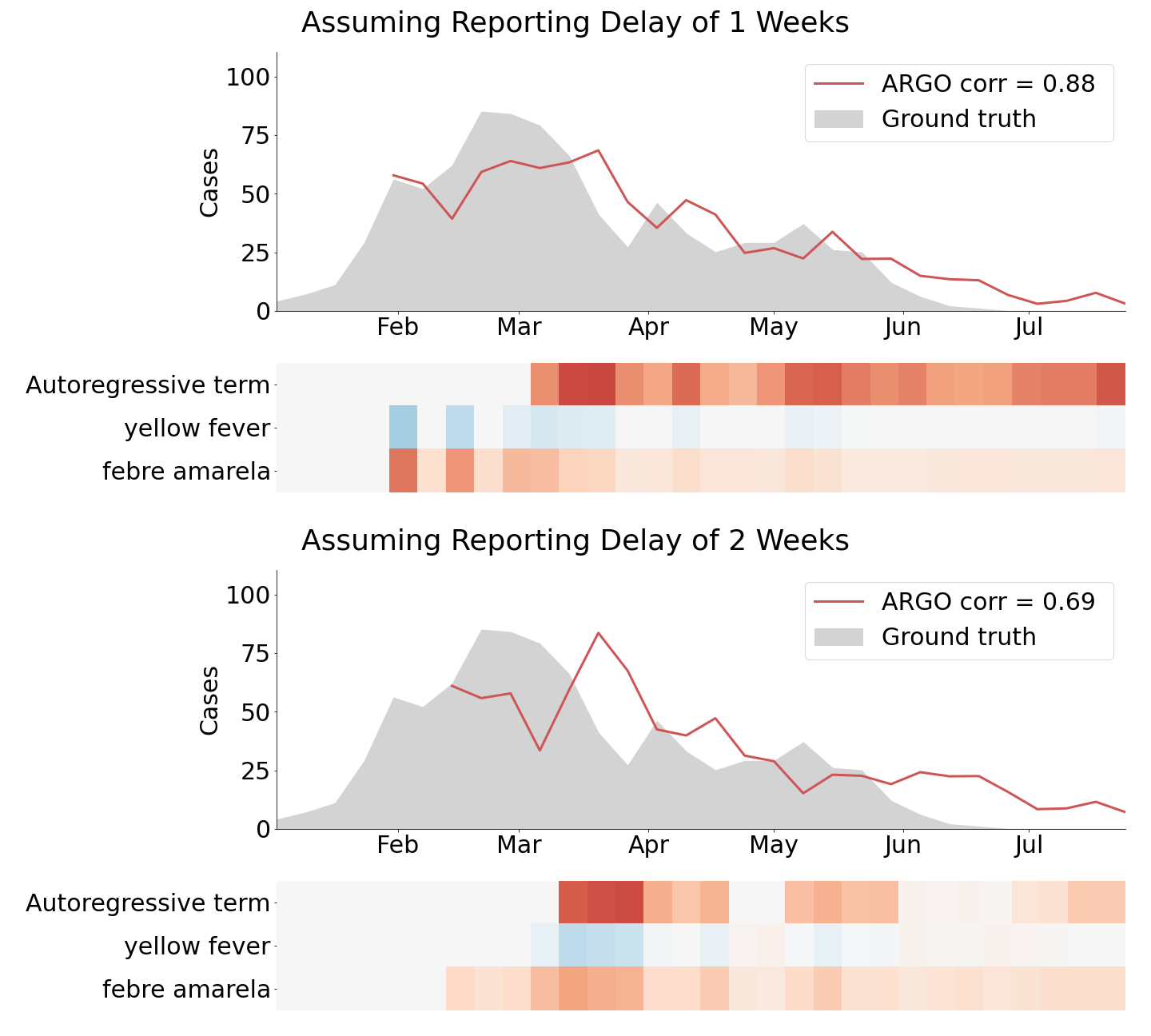

Supplement: S1 Fig — In the heatmaps, darkest reds correspond to largest positive coefficients and darkest blues correspond to largest negative coefficients; grey indicates a coefficient of zero. (TIF) [file pcbi.1008117.s001.tif]

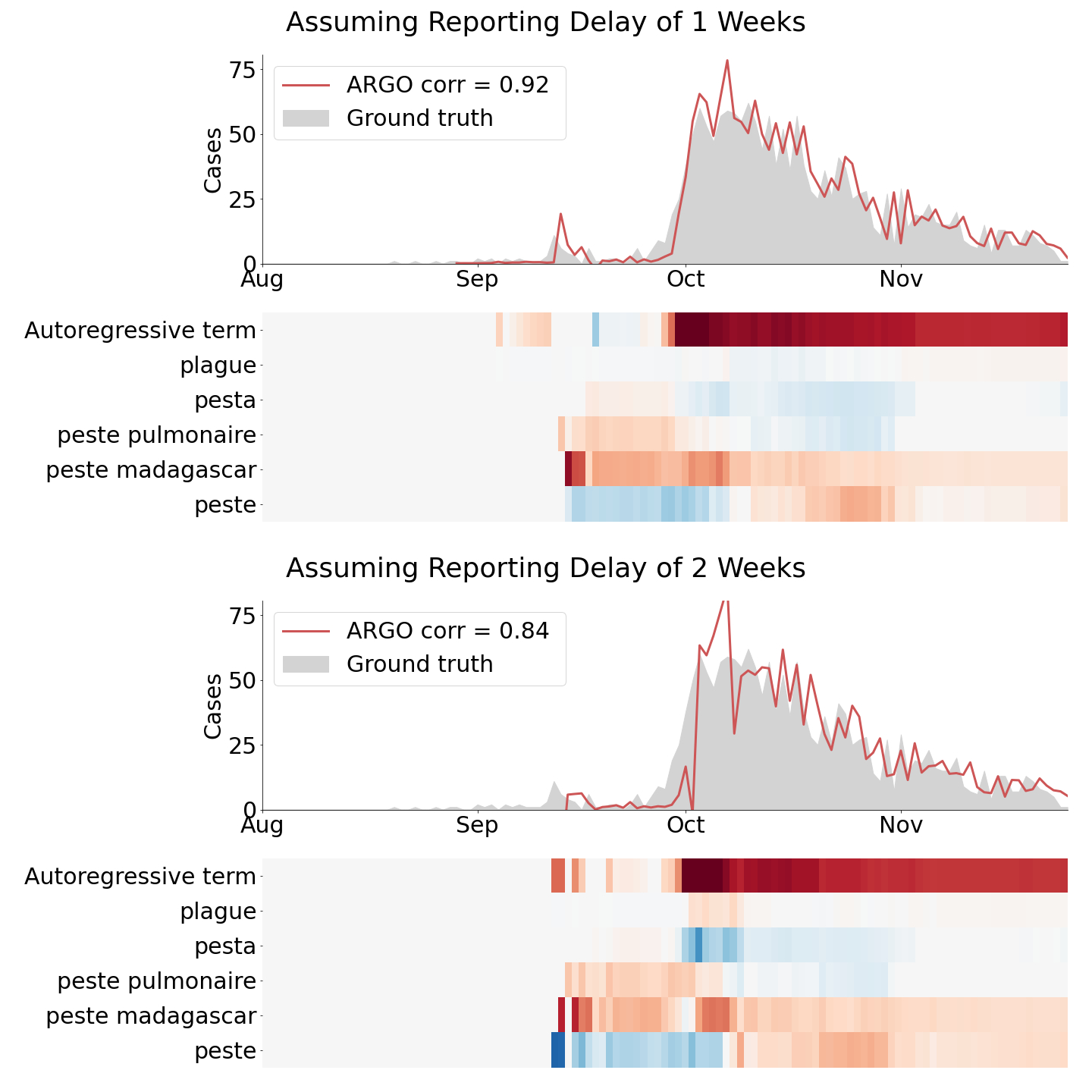

Supplement: S2 Fig — In the heatmaps, darkest reds correspond to largest positive coefficients and darkest blues correspond to largest negative coefficients; grey indicates a coefficient of zero. (TIF) [file pcbi.1008117.s002.tif]

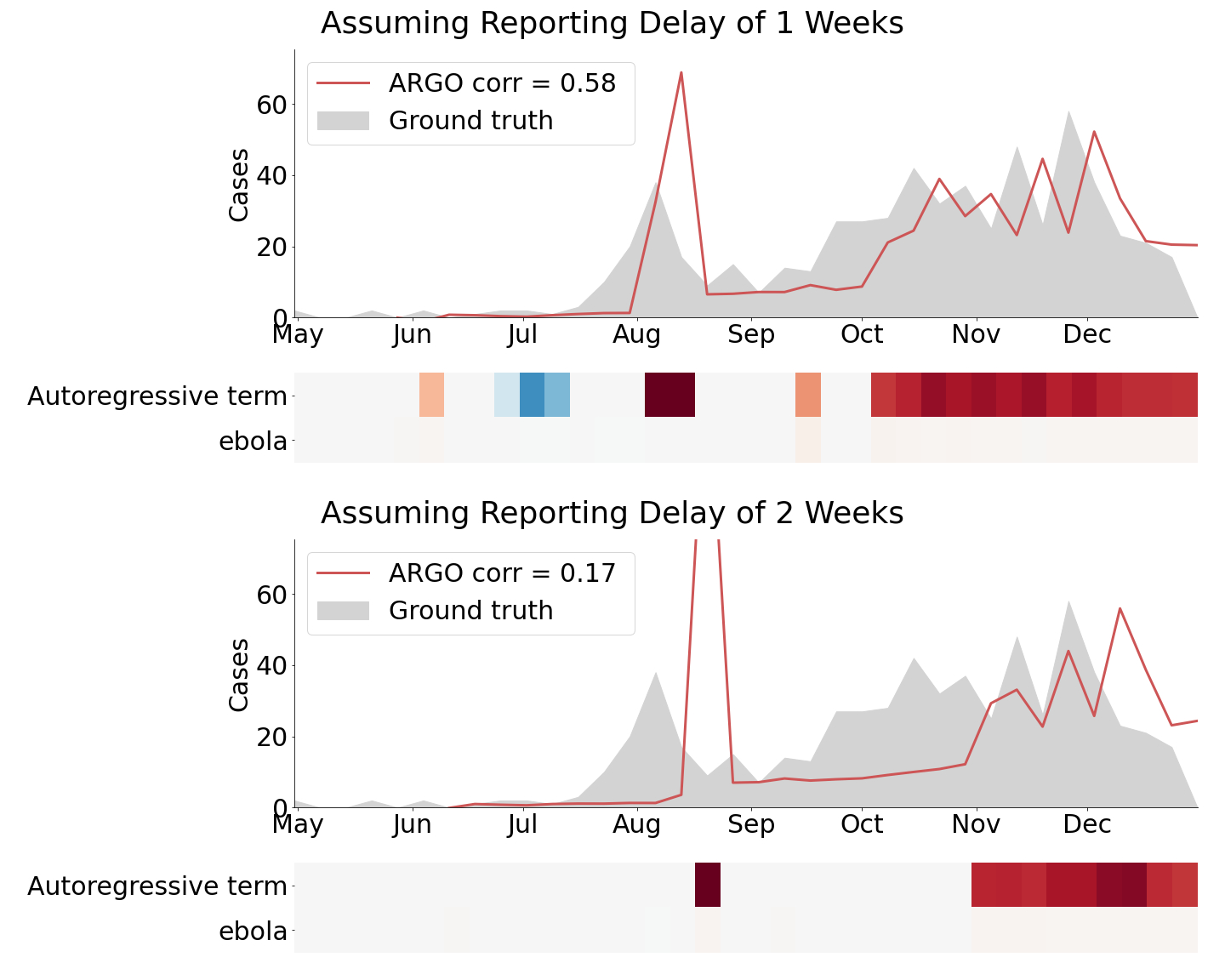

Supplement: S3 Fig — In the heatmaps, darkest reds correspond to largest positive coefficients and darkest blues correspond to largest negative coefficients; grey indicates a coefficient of zero. (TIF) [file pcbi.1008117.s003.tif]

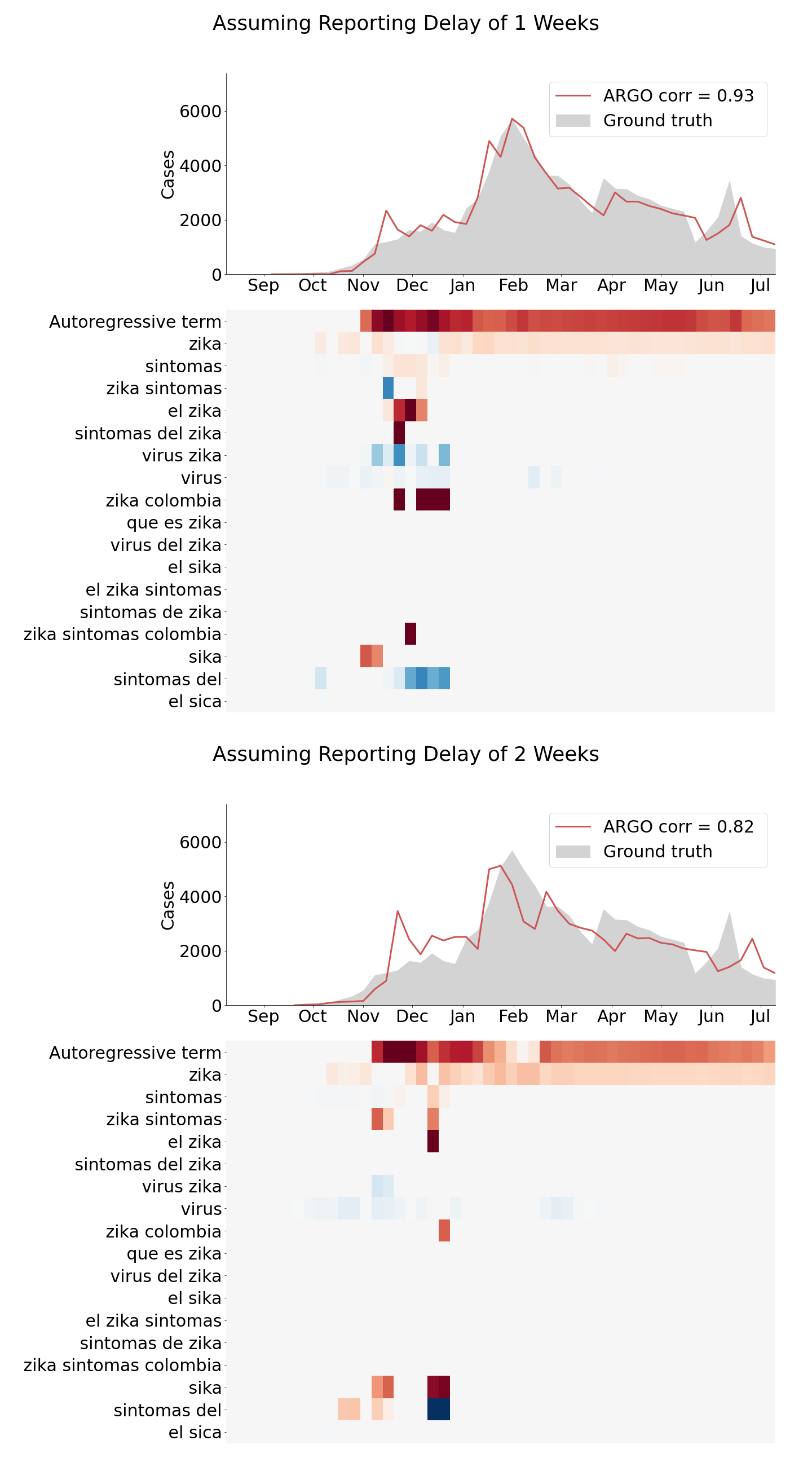

Supplement: S4 Fig — In the heatmaps, darkest reds correspond to largest positive coefficients and darkest blues correspond to largest negative coefficients; grey indicates a coefficient of zero. (TIF) [file pcbi.1008117.s004.tif]

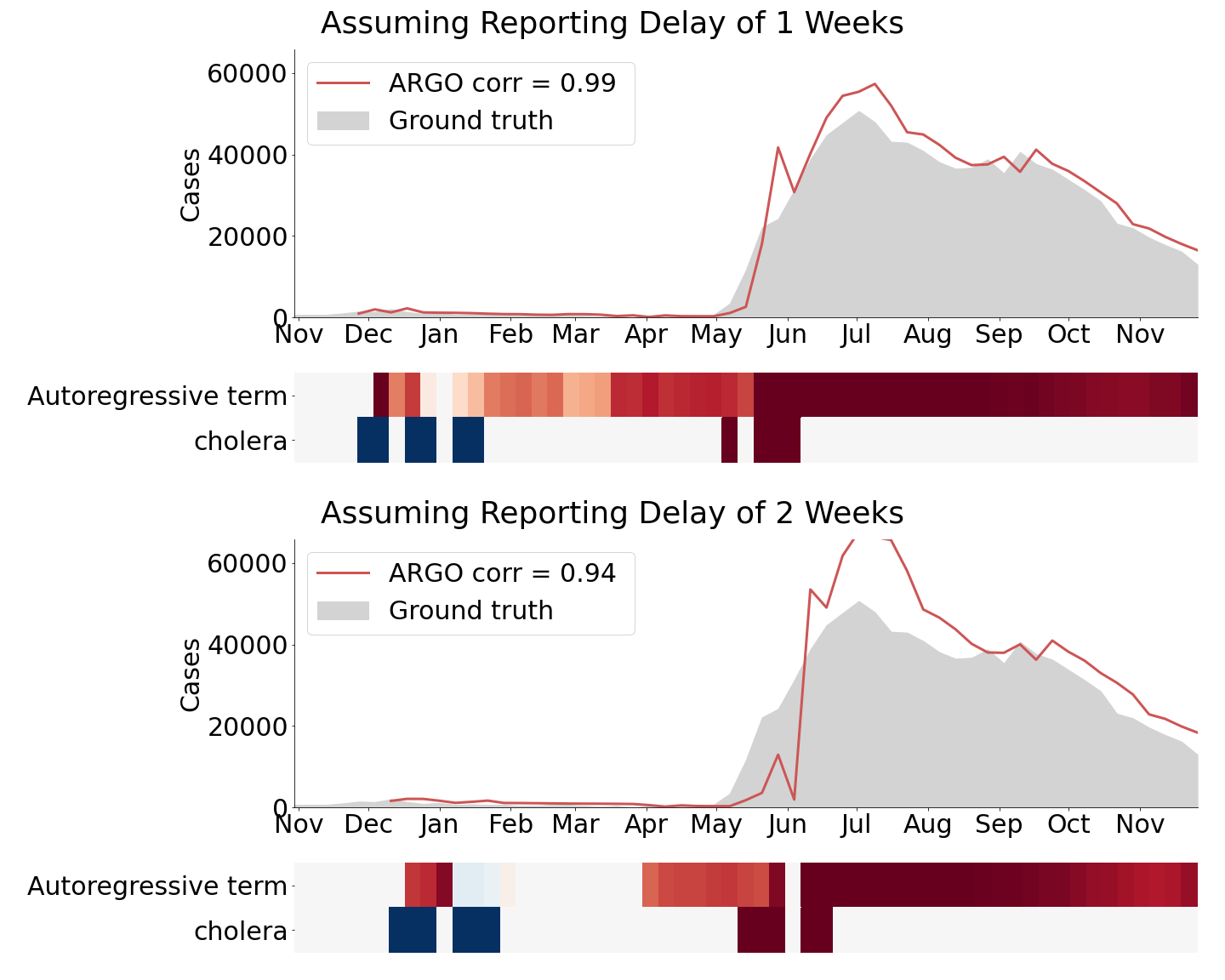

Supplement: S5 Fig — In the heatmaps, darkest reds correspond to largest positive coefficients and darkest blues correspond to largest negative coefficients; grey indicates a coefficient of zero. (TIF) [file pcbi.1008117.s005.tif]

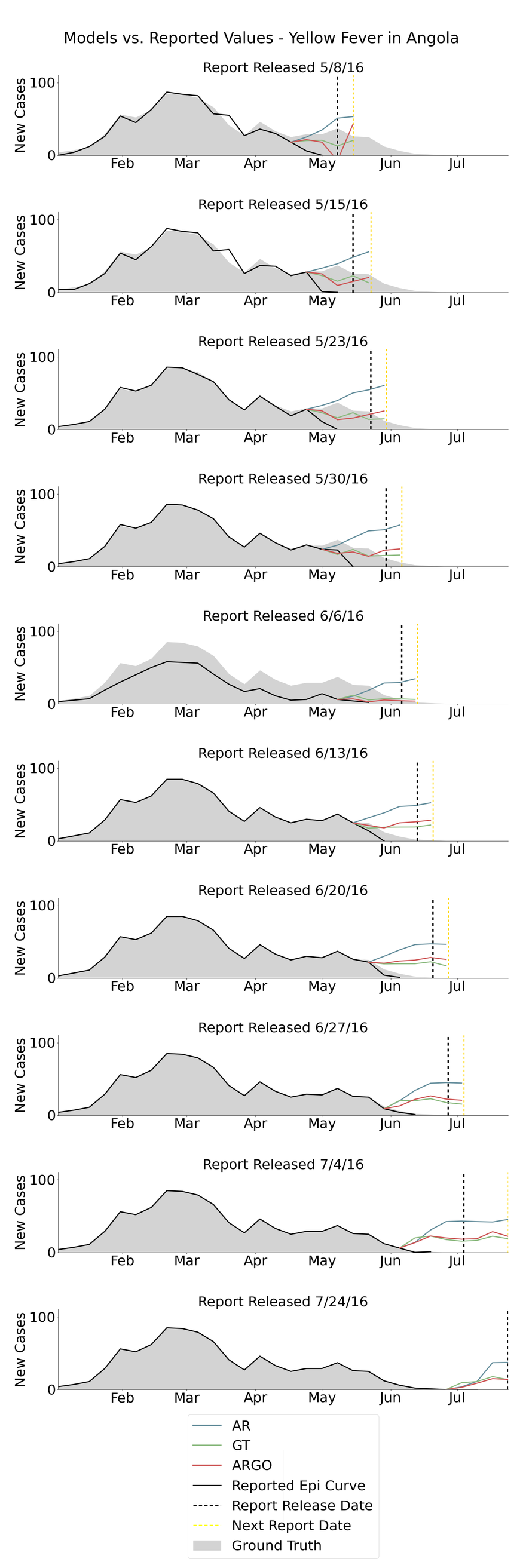

Supplement: S6 Fig — (TIF) [file pcbi.1008117.s006.tif]

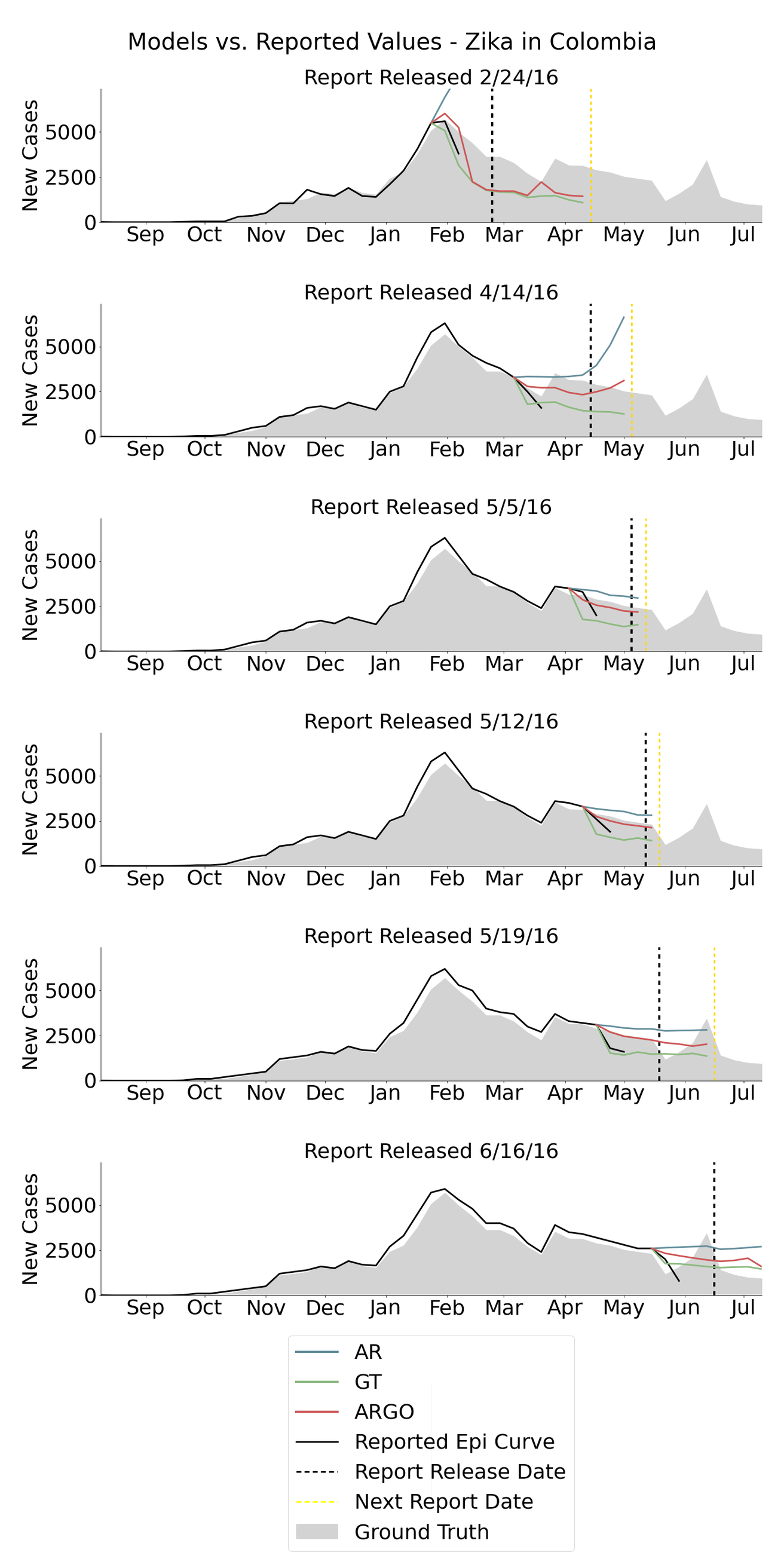

Supplement: S7 Fig — (TIF) [file pcbi.1008117.s007.tif]

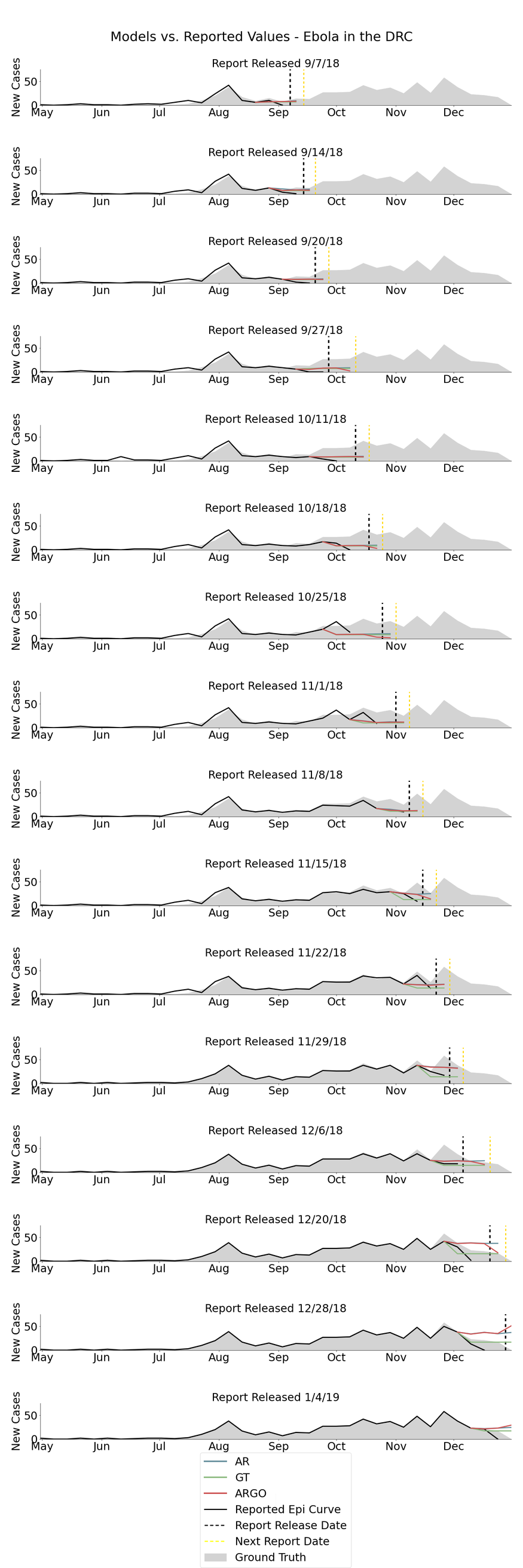

Supplement: S8 Fig — (TIF) [file pcbi.1008117.s008.tif]

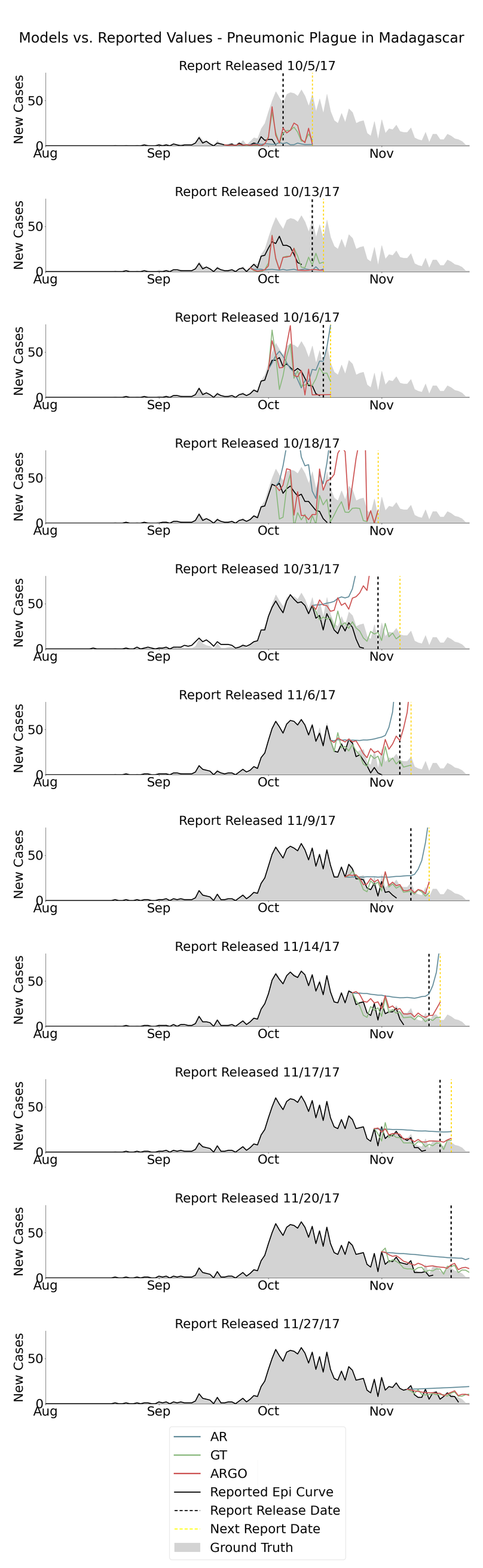

Supplement: S9 Fig — (TIF) [file pcbi.1008117.s009.tif]

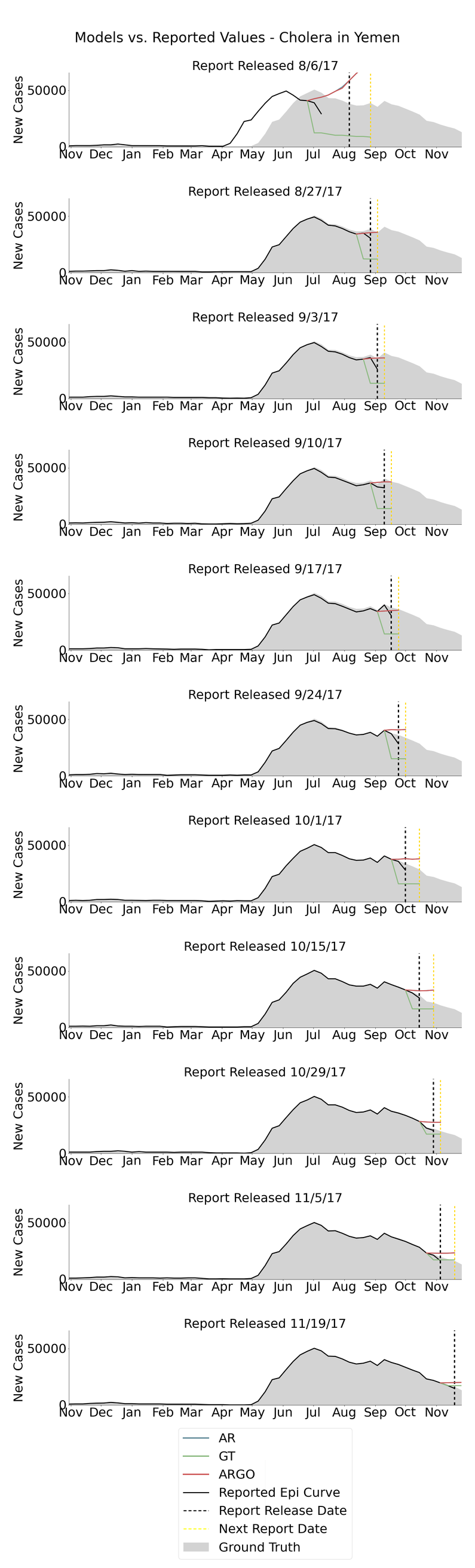

Supplement: S10 Fig — (TIF) [file pcbi.1008117.s010.tif]
