# Supplementary material for: Real-time estimation of disease activity in emerging outbreaks using internet search information
Source: PLoS Comput Biol. 2020 Aug 17;16(8):e1008117. doi: 10.1371/journal.pcbi.1008117 (PMC7451983; doi:10.1371/journal.pcbi.1008117)
Supplement: Attachment — Submitted filename: Outbreaks Paper Review Responses.pdf [file pcbi.1008117.s011.pdf]

Responses to Review Comments

We thank the reviewers for their constructive comments. We respond point-by-point to each comment in detail below, and have also submitted a version of the manuscript with changes highlighted alongside a clean version of the new manuscript. There were two broader concerns shared by multiple reviewers, which we briefly cover here as well as in detail below.

First, reviewers 2 and 3 both brought up the lack of quantification of model accuracy under the second evaluation regime (evaluation based on epidemiological reports released in real-time). We have addressed this concern by adding a new table, Table 2, with aggregate accuracy measures for the nowcasts of each model (as well as standard surveillance data) under this evaluation regime. We discuss the implications of the measures in this table in the results section (179-196), and describe the implementation of accuracy metrics in the methods section (lines 449-462). Second, reviewers 1, 2, and 3 all suggested additional discussion of the conditions that would facilitate effective use of these models, or at least conditions that could provide a "minimal use case" in which nowcasting models would be somewhat useful. We have expanded the discussion of these conditions -- including Internet coverage, disease transmission dynamics, serial interval, and signal-to-noise ratio in multiple data streams -- in the discussion section (lines 240-249, 254-265, and 309-320).

We respond below point-by-point to each comment. Note that line numbers refer to the version of the manuscript with changes highlighted; line numbers are slightly different in the clean version of the new manuscript.

__**Reviewer #1**__

1. *Throughout: Zika and Ebola are conventionally capitalized as they are named after places, but yellow fever, plague and cholera are not.*

   We have resolved the capitalization issue throughout the manuscript -- Zika and Ebola are now consistently capitalized, while yellow fever, plague, and cholera are not (except in titles of tables or figures).

2. *Line 75-76: At this point in the manuscript, it is not clear what is meant by "historic." I initially assumed that some kind of past outbreak data was used to inform a statistical model, but it seems the authors mean strictly data from earlier in the same outbreak.*

   We changed the wording here (now line 82) from "historic" to "autoregressive," to make it clearer that data from past outbreaks is not being used to inform a statistical model.

3. *Sentence spanning 77-80: A comma is missing between "countries" and "weeks", making it hard to read this sentence correctly.*

   We have added a comma to this sentence (now line 86).

4. *Line 91: What does "accurate" mean? I doubt we are talking about exact numerical values, and anything else is subjective/requires context.*

   We deleted the word "accurate" from this sentence (now line 98).

5. *Lines 100-105 and Discussion: The authors find that their methods vary in effectiveness. I find this understandable, but practically speaking it is problematic. How should a policy maker determine whether a nowcast is reasonable to use? Do the authors have any suggestions about how to predict the reliability of a nowcast, or how to characterize the settings where the methods are sane to apply? For context, I raise this point because the authors are clearly concerned with solving a real problem, and thus consider competing scenarios with high quality, regular surveillance data versus sporadic reports subject to revision (an experimental design choice I commend).*

We thank the reviewer for this comment in particular -- as they point out, the goal of this paper is to provide practical solutions that could be deployed to track contemporary outbreaks, and so addressing the practical use case is key. Here are a few thoughts on "more suitable" vs. "less suitable" use cases:

- In order for these methods to be effective, there must be a reasonable degree of internet coverage in the area in question. It is certainly not a requirement that everyone in the affected area must be equipped with an internet-enabled device -- but a non-negligible subset of the population needs to be.
- As noted earlier, diseases with shorter serial intervals seem to be easier to track with search queries than diseases with long serial intervals due to data quantity.
- Relatedly, diseases that affect large swaths of the population will be easier to track through trace data (for example, COVID-19 will draw large query volumes since it affects everyone).
- Perhaps most of all, these methods will be most applicable in outbreaks where there is little accurate ground truth data, and where any signal is better than none.

However, we hesitate to generalize across pathogens and populations to make sweeping assumptions about characterizing the settings where these methods best apply, as we only examine a small number of outbreaks here. We have augmented parts of the discussion (lines 240-249, 254-265, and 309-320) to attempt to walk the line of pointing out some of these hypotheses while calling for future work on characterizing the settings where methods based on search data are likely to be effective.

More generally, the reliability of any one source of digital trace data will be improved by augmentation with other data sources, whether ground-truth or another informal proxy measure. A multi-prong approach, rather than relying on a single data source, will be most robust to noise and biases -- an approach we look forward to exploring further in future work (for example, related papers evaluate nowcasting diseases on the basis of historical epidemiological data, internet search query volumes, social media data, crowdsourcing, and data from electronic health records -- Lu et al. 2018, McGough et al. 2017, and Santillana et al. 2015 have some good examples of multi-prong approaches). We have also added multi-prong approaches as a direction for future work (lines 309-316).

6. *Fig 1, possibly some other figures: I suggest not showing 1.25 on the y-axes, as that's an undefined value for data normalized on [0,1]. Also, these legends are highly redundant--they are all normalized to [0,1], so that should just be in the caption rather than twice in every panel; also, as each panel is labeled with the location, there's no need to state it twice in every legend.*

   We have changed Figure 1 to show only values up to one on the y-axis, and have changed the legends and caption according to the suggestion. We also ensured that Figure 5, which also has normalized values, displays only values up to one along the y-axis.

7. *Thought motivated by Fig 1: I don't believe the authors mention this, but it seems likely that populations have a kind of "search fatigue" or saturation effect. Early in an outbreak, there may be a heightened need for information about a disease, but unless looking for news updates, it seems that people would not keep searching for the same information. Relatedly, I would imagine there are meaningful trends in the types of search terms used, where people early on look for general information about a disease, how it's transmitted, etc., and later tend to look for information about symptoms and treatment. Is there any evidence for this kind of structure in search term data?*

   These are interesting points about the types of search terms expected to come up at different points in the outbreak and the types of people expected to be searching at different points in the outbreak. This comment also points to the greater question of search term selection. Due to the "data scarcity" of the environment in which each of these outbreaks takes place, we decided to go with a few search terms that are clearly related to the disease in question. We don't, for example, include search terms related to symptoms of the disease in question (though this would be an interesting direction for future work), so it is hard to say from this

paper's data which types of terms tend to be "trending" at the beginning vs. end of outbreaks. Other papers which use larger suites of search terms in more data-rich environments might provide a better basis for this analysis (see, for example, Lu et al., 2018). Better characterizing the types of searches and searchers that matter over the course of an outbreak is definitely an interesting direction for future research -- we have added a note of this idea in the conclusion, lines 266-274.

8. *Methods and Discussion: It should be stated that the final surveillance data here is taken as "truth" (and I don't think there's a serious alternative), although real-world reporting practices may vary in time.*

   We have now included this important point twice in the methods (lines 331 and 296) and a brief discussion of its limitations in the discussion section (lines 293-300).

9. *Fig 2: The late August spike in the Ebola/DRC nowcasting is somehow driven by autocorrelations in the AR and ARGO models. I understand why the nowcast peaks lag behind the ground truth data, and why the problem is worse for the 2-week lag than the 1-week lag, but what is it about this August peak that is so problematic for the autocorrelation model? I've looked at the methods, and it is not clear to me why this one part of the nowcasts would be so bad. Do the regressions normalize by current size of the outbreak? An increase from 1 to 3 cases from one week to the next is less meaningful than an increase from 100 to 130, but perhaps the AR for Ebola/DRC is "learning" too much from the 1.5 months of basically nothing at the beginning of that time series. The lag between the ground truth data and the nowcasts seems to be roughly double the reporting delay, but mysteriously only for Ebola/DRC--plague/Madagascar is on a similar scale ([0,~50]), but the predictions are much better. I would appreciate if the authors would dig into this a bit more.*

   Models do not normalize by the current size of the outbreak, so your hypothesis regarding the AR model "learning" too much from early on in the Ebola epidemic is likely correct. One potential way of dealing with these issues is to introduce a shifting training window or weighting more recent training data more heavily, as suggested in another comment from the same reviewer (but, as noted in the response to that comment, there is an inherent trade-off between restricting or weighting training data, and taking advantage to the highest degree possible of the limited data we have available). The Plague outbreak in Madagascar is somewhat unique among the outbreaks explored here because ground-truth epidemiological data are available at a daily granularity (see table 2). Therefore, while epidemiological reports from the plague outbreak exhibit similar release delays to other outbreaks (around two weeks), they do provide significantly more training data, which could explain why we do not see an over-prediction of the peak cases as in the Ebola outbreak. We have addressed the question of training windows and data scarcity more thoroughly in the methods section, lines 414-417.

10. *Fig 3, and other figures: There is no legend for the heatmaps, nor is the meaning of the colors stated anywhere in text.*

    We have added a description of the color coding for the heatmaps to the caption of Figure 3 as well as Figures S1 - S5.

11. *Line 171: I have no idea what "within-range" means. "Meaningful" is subjective, but at least it sounds subjective. Unstated, I think, is that the authors are applying some human intuition for what kind of accuracy would be required for appropriate public health responses. They probably should say that this is what they're doing (or explain what alternative criteria are being applied). Also, I think the word "that" is missing between "estimates" and "filled".*

    We have made the evaluation of model accuracy in this second evaluation paradigm more concrete by adding a table (Table 2) that compares the accuracy of each model to the accuracy of the reported epidemiological cases released in situation reports. The table compares the accuracy of models to those of reported epidemiological data in the second-to-last and last week before epidemiological data ceases to be

available, and also provides the accuracy of models in the first week after epidemiological data is available as a point of comparison. The table aggregates errors by taking the mean across all epidemiological reports for each outbreak (N = 7-17). In this table we evaluate the percent error for each model in the second-to-last week that data is reported in each situation bulletin, the last week that data is reported in each situation bulletin, and the first week in which data is not reported in each situation bulletin. The results of the table highlight that ARGO is typically the most accurate of the three models by either measure, and frequently more accurate than reported epidemiological data. We also present these results briefly in the text prior to the table, lines 178-188.

12. *Throughout: The space between "Fig." and the number is too big. If this is Latex, the spaces aren't being escaped properly, e.g. should be "Fig.\ 3". Same for e.g. "Jan.\ 3".*

    Spaces are now escaped properly after abbreviations throughout the manuscript.

13. *Lines 215-217: I do not have any intuition for why GT data would be more useful for diseases with short serial intervals. Do the authors have any idea why this might be the case?*

    Our hypothesis is that in diseases that spread quickly and affect large swaths of the population, there is in general more data -- both ground truth epidemiological data and trace data from Internet searches -- available, so there is a higher signal to noise ratio, and models perform better. Diseases with longer serial intervals, which spread more slowly, will naturally result in scarcer data from all sources. We have added a brief discussion of these questions in lines 2342-249.

14. *Line 221: result, not results*

    This issue has been resolved (now line 247).

15. *Throughout: Inconsistent capitalization of Internet.*

    The word Internet is now capitalized throughout the manuscript.

16. *Line 228: Some explanation of how search terms are chosen should be mentioned here, or earlier in the results. This is a very important issue, and shouldn't be buried in the methods.*

    We have added a few sentence discussion of search term selection to the discussion section (lines 266-274). We also now include descriptions of search term selection in the results section (lines 130-131) and the methods section (lines 342-358).

17. *Lines 242-250: Can media coverage be used as a predictor (aka feature in ML jargon)? I can understand why the authors might not want to use media attention as a predictor of what's happening on the ground, but it seems like it could be very useful as a correction term for search activity. In other words, an increase in search activity is more meaningful if it does not coincide with an increase in media coverage. A locale-specific relationship between media coverage and search patterns could be determined during non-outbreak periods, or for an unrelated health problem.*

    We are excited about the idea of potentially using media coverage as a predictor in a more holistic ML model for nowcasting cases based on historic epidemiological data and a suite of "digital trace" data sources available in real-time. Specifically, as this comment suggests, it is possible that we could to some degree subtract the signal from media from the search trends signal to uncover a "true" search trends signal that represents the volume of users searching for information on a disease free of bias introduced by media coverage. We would like to see this explored in future work, and have added a note of this idea in the conclusion in a more general discussion of multi-pronged approaches (lines 314-316).

18. *Lines 298-301: Is Google universally the preferred search engine? Baidu in China? Is Google what Arabic speakers would principally be using?*

    Differences in language and search engine preferences across cultures are key -- we have added this point as a limitation in the conclusion (lines 262-265).

19. *AR model in Methods: If I understand correctly, at each time point, a linear regression is constructed using all past data to estimate the coefficients, but only the most recent observation (y_t-h) is used as an input. This means that having a long history of small, noisy values would result in a model that has a beta of roughly 0. If more recent data is not weighted as more informative than observations farther in the past, a dramatic increase might still be treated as uninformative, even after a couple such observations. If I am not understanding this, please clarify. In any case, what is going on with the AR model seems relevant to the Ebola/DRC prediction problems and warrants more discussion.*

    Certainly, the training "lookback window" is an essential parameter to consider tuning when working with these autoregressive models and/or autoregressive-plus-digital-trace-data models. The original paper proposing ARGO as an influenza-tracking model in the United States looks closely at this question, selecting a 2-year training window (so at each time-step *t*, the model is trained on data from time-steps *t-104...t-1*) (see Yang et al. pg. 3). Our paper deals with a significantly more data-scarce environment, where models are trained on anything from three weeks of weekly data (N = 3) to, at most, a handful of months of weekly data (N < 20). Although there is an inherent tradeoff between using small training windows for local specificity vs. larger ones for model convergence, we chose to use all data available for training here due to the overall data scarcity (though, as you point out, this could result in a beta of roughly 0 if there is a history of small, noisy values). We've added a shortened version of this description to the methods section, lines 414-417.

20. *Fig S2: Why are there more rows in the heatmaps than there are row labels?*

    Figure S2 has been enlarged so that labels are visible for all rows in the heatmaps.

21. *References: These need to be cleaned up. There are random spaces in URLs, and inconsistent/incorrect capitalization.*

    References have been cleaned up and formatted with PLOS Computational Biology's bibtex style file.

22. *A final thought: As perhaps the most famous (and now aborted) example of nowcasting, Google created Flu Trends and Dengue Trends. It might be appropriate to mention that effort in the introduction.*

    We have added a discussion of GFT and GDT in the introduction section, lines 74-80.

Reviewer #2:

1. *Perhaps my greatest concern is the lack of quantification of the performance of the authors' forecasters in the subsection "Evaluation Based on Publicly Released Reports". This is clearly the scenario of greater interest, and although one can see from the figures that the forecasts are reasonable, it is difficult from figure 4 to see that "ARGO appears to most closely estimate the cases that would eventually be reported throughout each outbreak," as the authors write on line 173. A table comparable to Table 1 for this scenario seems like it could provide much clearer support for such a statement. Furthermore, the inclusion of a quantitative metric would allow for later work to easily be compared with this work. On line 381, the authors seem to indicate that such quantification was not possible due to a lack of ground truth data, but the figures invite visual comparison of the forecasts with a ground truth time series, so I find that statement of the authors confusing.*

    We thank the reviewer in particular for pointing out this key quantitative analysis that was missing from our original manuscript. We thought more about how best to quantify the results presented in Figure 4 and Figures S6-S10 in terms of aggregate accuracy measures. We have now made the evaluation of model

accuracy in this second evaluation paradigm more concrete by adding a table (Table 2) that compares the accuracy of each model to the accuracy of the reported epidemiological cases released in situation reports. The table compares the accuracy of models to those of reported epidemiological data in the second-to-last and last week before epidemiological data ceases to be available, and also provides the accuracy of models in the first week after epidemiological data is available as a point of comparison. The table aggregates errors by taking the mean across all epidemiological reports for each outbreak (N = 7-17). In this table we evaluate the percent error for each model in the second-to-last week that data is reported in each situation bulletin, the last week that data is reported in each situation bulletin, and the first week in which data is not reported in each situation bulletin. The results of the table highlight that ARGO is typically the most accurate of the three models by either measure, and frequently more accurate than reported epidemiological data. We also present these results briefly in the text prior to the table, lines 178-188.

2. *My next greatest concern is the interpretation of the size of the L1-penalized regression coefficients as variable importance in Figure 3 and similar. In my experience, with small data sets such as those analyzed by the authors, the value of these coefficients can be highly sensitive to the random choice of the folds used for cross-validation. Also, the choice of the folds for a time series application where there is a clear temporal correlation structure deserves some discussion. From the code, it seems that the temporal structure of the data is ignored in the choice of folds. How do authors justify this and what effect do they anticipate this has on the selected regularization parameter? Finally, since many of the linear model predictors are likely highly correlated, I think an elastic net penalty would be more appropriate than a lasso penalty if the authors would like to draw some conclusions about the relative importance of the different variables. Zou and Hastie (2005) have shown that the lasso penalty can result in one variable in a correlated group randomly getting a large coefficient, whereas the elastic net penalty can lead to a more equal size of coefficients among members of the group.*

The reviewer is correct to point out the instability of chosen coefficients using LASSO. This broad observation on the LASSO methodology applies to cases when large sets of highly collinear groups of variables are used as input. In our models, the number of input variables is small (two for Ebola in the DRC and Cholera in Yemen, to three for yellow fever in Angola, six for plague in Madagascar, and eighteen for Zika in Colombia). The collinearity between the inputs is not extremely high (except in cases like yellow fever in Angola for which there are very few input variables), thus the "condition number" of the regression problem (a proxy for when the choice of coefficients in LASSO will be unstable) is not that high, so instability in LASSO should not be too much of a worry. The plots at right show the correlation between search term time-series for the three outbreaks for which we use multiple search terms.

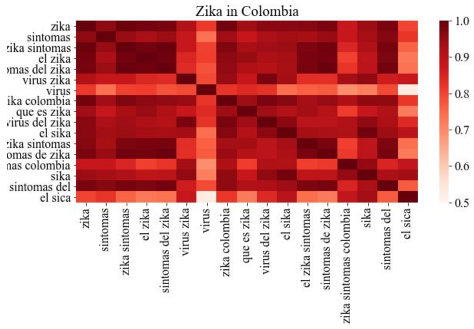

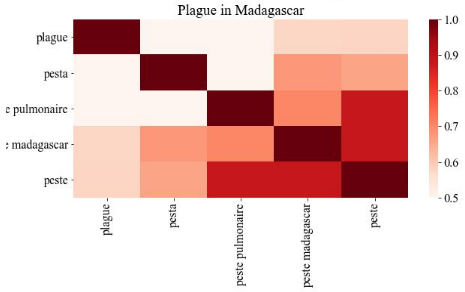

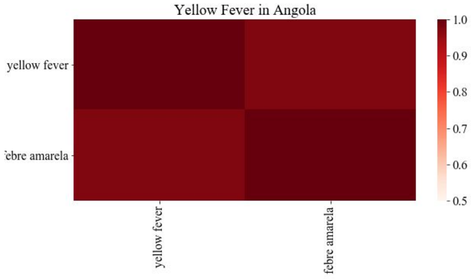

The reviewer is correct, also, that in cross-validating to choose the LASSO penalty parameter the time-series structure of the data is ignored (and we appreciate the time taken to look at our code!). Other papers using ARGO in data-rich environments have resolved this issue somewhat by training only on recent data (e.g. using a sliding training window) so that only recent data is used for penalty

parameter selection (see, for example, Lu et al., 2018). We have chosen not to use a sliding training window or a “fancier” time-series cross validation technique for selecting the penalty parameter given the scarcity of data for training (particularly early in outbreaks, only a few data points are used for training and cross-validation, and even at the end of outbreaks time-series are still very short compared to relatively data-rich applications like influenza).

We would like to continue using LASSO over elastic net for consistency with past work using ARGO-like models to nowcast other diseases in other contexts. We would also like to address the reviewer’s concern, although as noted above we believe it theoretically does not apply to a great extent to our use case. In order to address the concern empirically, we have adjusted our procedure to allow for better interpretation of coefficients in each LASSO model: rather than producing coefficients with one model run, we run each LASSO ten times with ten different random seeds, and use the average of the coefficient associated with each input feature over the ten runs as a measure of the importance of that feature. Figures 3, S1-S5 in the new version of the manuscript use this new procedure, and the procedure is described in the Methods section, lines 390-392. The new figures do not look different from the old figures produced with just one model run, confirming our earlier hypothesis regarding model stability due to the low condition number.

3. *Figure 4: The dates on the top of the panels do not always align with the vertical reference lines. For example, consider the panel in the upper left corner.*

   This issue has been resolved for all panels in Figure 4.

4. *Figure 4 caption: Figs. S9-12 should be Figs. S6-10?*

   Yes -- we have changed this to Figs. S6-10.

5. *Line 215: "we find that GT data appears to possess greater predictive power in diseases with short serial intervals like influenza, and less predictive power in diseases like Cholera, where transmission time-scales are typically longer." The supporting evidence for this statement is unclear.*

   Our hypothesis is that in diseases that spread quickly and affect large swaths of the population, there is in general more data -- both ground truth epidemiological data and trace data from Internet searches -- available, so there is a higher signal to noise ratio, and models perform better. Diseases with longer serial intervals, which spread more slowly, will naturally result in scarcer data from all sources. But as you point out, this is just a hypothesis, based on an analysis of only five outbreaks here (along with the literature on influenza nowcasting). We have expanded our discussion of the hypothesis in the discussion (lines 244-249), as well as pointing out the limitations of forming this and other generalizations from only a handful of outbreaks and the need for future work on additional outbreaks (lines 309-320).

6. *Line 314: "The time horizon of prediction h depends on the reporting delay in each outbreak" It is unclear to me why the authors link the prediction horizon and model lag in this manner. I would consider it simpler to use a lag-1 term in all models and projecting forward multiple steps when necessary for prediction.*

   Choosing a lag-1 model and recursively projecting forward is known to be a bad choice in autoregressive modeling. This choice introduces amplification (resonances) in the system that lead to (catastrophic) exponential error growth as the time horizon of prediction increases.

   Think of the following lag-1 model:
   $y(t) = a * y(t-1)$

   Thus,
   $y(t+1) = a * y(t) = a^2 * y(t-1)$

and

$y(t+n) = a^{(n+1)} * y(t-1)$.

Therefore, for any $|a| > 1$ and $y(t)$ different from 0, $y(t+n)$ grows exponentially as n increases, independently of the $y(t-1)$ for large enough n, or converges rapidly to 0 if $|a|<1$.

7. *Equations (2) and (3) make use of variables that the authors never define and do not seem to accurately represent a linear model.*

   We have added text to define the variables used in equations 1, 2, and 3 (autoregressive term y_{t-h}, synchronous incidence y_t, and the set of synchronous Google query volumes $G = \{g_1, ..., g_k\}$. We believe that these equations do represent a linear model.

Reviewer #3

I applaud the authors for tackling the difficult problem of what to do in the context of inaccurate surveillance reports, particularly for diseases new to specific geographic areas (or new altogether). Current events are only the most recent reminder of how unfortunate delays can be. The paper is relevant, well-written, and the examples provided are comprehensive. Below are some suggestions for improvement.

Lines 171-172: Is there a word missing here? Please check.

This sentence was deleted in the newest version, so it is no longer an issue.

The severe delays in getting even preliminary reports out is on painful display in the examples provided (Figure 4). Each epi curve in that figure has a 'report released' line and a 'next report released' line, but the 'next report released' timing does not align with the following epicurve's 'report released' line. I see the supplemental files with all of the reports, but how did the authors choose which to present in the main paper?

Many of the outbreaks we tracked did not release any reports during the main body of the outbreak (see, for example, yellow fever in Angola). We chose to include reports in Figure 4 that occurred during the main body of the epidemiological curve while changes in disease dynamics were rapid, rather than including reports from long before the outbreak occurred or during the "long tail" following the outbreak. Within the main body of the outbreak, we chose evenly spaced case reports. We hope that Figure 4 will act as a "teaser" encouraging the readers to look at all case reports in the supplemental information. We have added a briefer version of this discussion to the caption of Figure 4. We have also added an associated Table 2, which displays aggregate accuracy measures across all case reports for each outbreak.

Were the authors able to make some kind of quantitative measurement of the amount of 'back corrections' that appeared in consecutive reports and were the magnitude of 'misreports' associated with the model performance?

The newly-added Table 2 quantifies the error of situation reports in the second-to-last and last week that epidemiological data is available (we report the mean and standard deviation over all situation reports for each outbreak, N = 7-17). In many outbreaks, the percentage error is quite high (for example, for Yellow Fever in Angola, the 2nd-to-last reported case value is typically 67% off from the ground truth, and the last reported case value is on average 81% off from the ground truth). We do observe that model performance is generally worse when the error in reported epidemiological data is high -- this is intuitive, because models trained on a less reliable ground truth are likely to have lower predictive accuracy than models trained on a reliable ground truth. This points to your last comment, regarding the importance of better traditional epidemiological surveillance -- we have made a note of this in the conclusion (lines 296-300).

Lines 196 – 200: The authors propose a minimal case use for these models – that they could at least signal whether the outbreaks are increasing or decreasing, or 'over' or 'not'. Can the authors quantify in the results the number of times each model got this right when the report got it wrong? Some kind of quantification to justify this claim would be helpful. It's clear that it happened in one case, but some additional evidence that this is a common occurrence would be useful.

This particularly helpful comment encouraged us to clarify what we mean by "over" or "not." We've changed the language in this sentence (now lines 222-228) to be more nuanced: the important point is that epidemiological reports frequently display a down-turn at the end of the epidemiological curve due to underreporting, implying that the outbreak may be coming to an end. Since the models do not suffer from the underreporting issue, they exhibit no such downturn, frequently suggesting that the outbreak is continuing when the most up-to-date epidemiological data suggests otherwise. This phenomenon appears to some degree in all outbreaks, but is particularly visible in the Yellow Fever data from Angola (see figure 4, Yellow Fever, panel 2), the Ebola data from the DRC (see figure 4, Ebola, panels 1-3), and the plague data from Madagascar (see figure 4, Plague, panel 3).

Also, it's not clear in the methods, but is the assumption that the forecast would be available on the day that the reports came out? Is there any data entry or analysis time included in the assumptions of when forecasts would be available or is it reasonable to assume these are instantaneous?

We assume that data entry and analysis time are instantaneous, as these algorithms could be run with the proverbial "click of a button" once the system was in place. We have added a note of this in the methods, lines 445-447.

The authors should consider making a plug for investments in better traditional epi surveillance, particularly for emerging threats. Models are very helpful, but only once we identify outbreaks and have some data coming in. The findings showing that even incomplete reports could yield useful model results is a strong rationale for releasing data as early as possible. Early data release remains a barrier to protecting global health.

We added a discussion of this importance of better standard surveillance and early release of case reports to the discussion, lines 296-300.
